# Longitudinal study of endodontic and periapical status of an adult Danish population examined in 2009, 2014, and 2019: a repeated cohort study

**DOI:** 10.1080/00016357.2023.2268699

**Published:** 2024-03-22

**Authors:** Ankur Razdan, Lars Schropp, Michael Væth, Lise-Lotte Kirkevang

**Affiliations:** a Section for Oral Radiology and Endodontics, Department of Dentistry and Oral Health, Aarhus University, Aarhus, Denmark; b Department of Public Health, Aarhus University, Aarhus, Denmark

**Keywords:** Ageing, apical periodontitis, longitudinal studies, root canal therapy, tooth loss

## Abstract

**Objective:**

To describe endodontic changes in an adult Danish population (C2;2009–2014–2019) and compare them with a similar cohort (C1;1997–2003–2008).

**Material and methods:**

A randomly selected cohort (C2) with three full-mouth radiographic examinations. The frequencies of teeth, apical periodontitis (AP), root filled teeth, and lost teeth in C2 were compared to a similar cohort (C1) using regression analyses; effect of age, cohort, and period was assessed.

**Results:**

C1 had 330 and C2, 170 participants (mean age, C1: 42.9; C2: 47.3years, *p* <.001). The proportion of individuals with no AP was similar in C1 and C2 (*p* =.46). C2 had a higher proportion of individuals with no root filled teeth (*p* <.001) and no tooth loss (*p* =.02) than C1. The proportion of AP and root filled teeth increased with age in both cohorts. C2 had fewer root filled teeth and lost teeth, fewest lost teeth in the youngest age groups.

**Conclusions:**

In C2, the prevalence of teeth with AP and root fillings increased with age, and few teeth were lost. Change in proportion of AP was similar in two cohorts; fewer root filled teeth and lost teeth in C2. The proportion of lost teeth in C2 showed cohort effect for older age groups.

## Introduction

Apical periodontitis (AP) has a widespread prevalence in the global population [1] and is prevented or treated by performing root canal treatment. The status of AP and root filled teeth impact the survival of teeth [2], and tooth retention is relevant for oral health-related quality of life [3].

The global proportion of elderly has been increasing significantly [4]; individuals are living longer and tend to retain their natural teeth [5,6]. With more teeth present during old age, there is a risk for the development of dental disease, and this may further increase the risk of dental treatments, such as root canal treatment, and of tooth extraction [7]. It is therefore relevant to monitor and evaluate the development of AP and the effect of treatment, as individuals grow older. This will provide a comprehensive understanding of the effects of ageing, and thus aid strategic planning of preventive and treatment resources to meet the future requirements within the elderly population.

Disease incidence and prevalence, as well as treatment strategy and outcome, may change over time. Different factors are responsible for these changes and in epidemiology age, period, and cohort effects have been used to describe such time related changes [8]. **Age effects** refer to the changes occurring in an individual as a consequence of getting older. A well-known example of an age effect is the development of gray hair. **Period effects** refer to changes, which occur due to external factors occurring over time. Such changes will affect all individuals being exposed to the factors, for example the effect of a pandemic, a war, or a global financial crisis. **Cohort effects** are effects, which relate specifically to the time of birth; an example could be the effect of the use of thalidomide in the 1950s as a harmless drug against nausea during pregnancy but causing severe malformations of the fetus. Age, period, and cohort effects are interrelated, and therefore it can be difficult to estimate if a change in individuals of a population is caused by one of the factors or a combination of these effects.

Longitudinal cohort and repeated cross-sectional studies may be used to describe and understand different aspects of changes in individuals and in the general population, respectively. In longitudinal cohort studies, a defined group of individuals is followed over a period of time – where individuals get older, and time passes – by performing repeated examinations and evaluations of changes in disease and treatment status [9]. Categorizing the study population into age groups at baseline allows evaluation of changes within each age group and comparison of changes occurring between different age groups [10]. A longitudinal cohort study of individuals in an adult Danish population analyzed the development of endodontic and periapical status and tooth retention from 1997 to 2008 (Cohort 1: C1). This population-based cohort study showed that the proportion of teeth with AP in an individual increased with age, irrespective of the time period [10].

Repeated cross-sectional studies compare the status of individuals from two similar study populations at different time points and thus assess the overall time-related trends in the population. A repeated cross-sectional study based on baseline information from C1 and Cohort 2 (C2) has previously been performed [11]. Overall, the study showed that individuals in C2 had fewer root filled teeth than in C1, however, the periapical status of root filled teeth did not differ between the cohorts. C2 has also been reexamined after 5 years and again after 10 years and a repeated longitudinal cohort study of the root filled teeth in the two study populations has recently been published [12].

The present study considered all teeth in the two cohorts. The aim of this study was to identify changes in endodontic and periapical status that may be attributed to changes in age, period, or cohort using radiographic data from individuals in an adult Danish population examined in 2009, 2014, and 2019 (C2), and to compare these data with findings from a similar cohort examined in 1997, 2003, and 2008 (C1).

## Materials and methods

### Study design and population

This population-based longitudinal observational study relied on the follow-up of a randomly selected cohort of individuals, who were followed over approximately 10 years (C2). The study design was similar to a longitudinal cohort study of the adult Danish population (C1), which was described in a previous article [10].

### Study population C2

**Figure 1:**
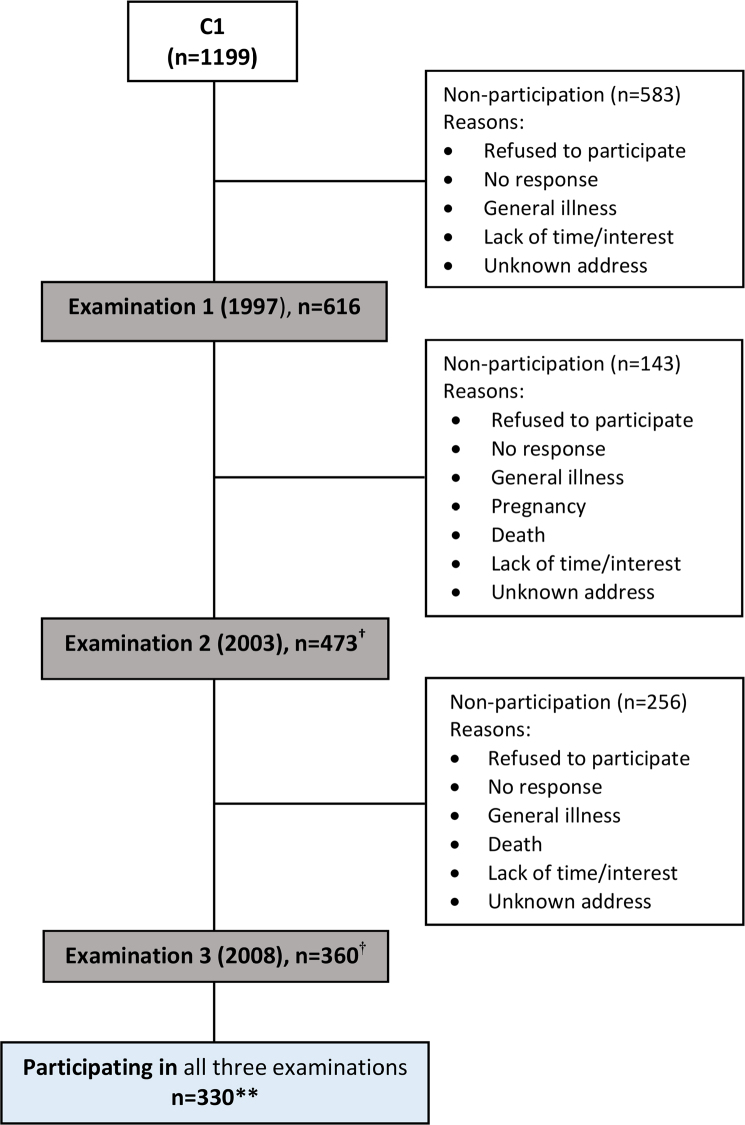
Flowchart illustrating participation of individuals in C1. *C1: Cohort 1 (1997–2003–2008), detailed information in Kirkevang et al. [10,16]; †Participants from Examination 1 were invited to participate in Examination 2 and 3; **Delayed registration for three participants explains discrepancy in total number of participants reported

**Figure 2:**
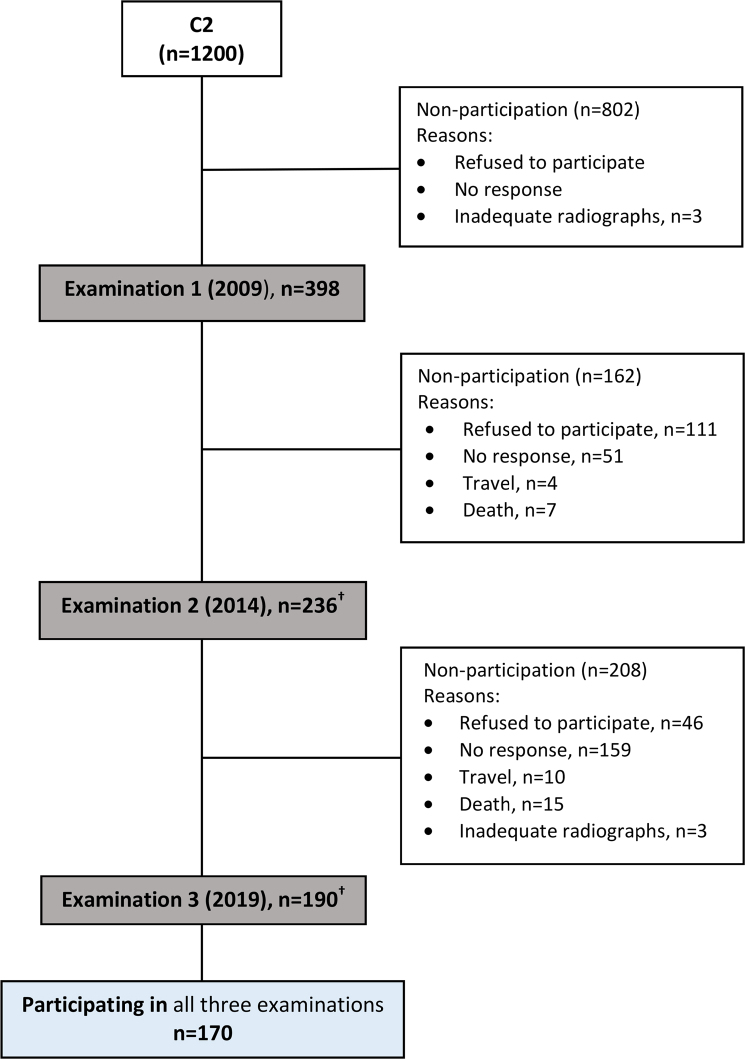
Flowchart illustrating participation of individuals in C2. †Participants from Examination 1 were invited to participate in Examination 2 and 3. C2: Cohort 2 (2009 − 2014 − 2019)

A random sample of individuals aged 20–64 years was systematically drawn from the general, adult population in Aarhus County, Denmark, from the Danish ‘Civil Registration System’ (CRS) in 2007, using birth dates and the year of birth as extraction keys. Initially, 1200 individuals were selected and contacted. The individuals were sent written information about the project and invited to participate. If the individuals did not want to participate, they were asked to give the reason for refusing. In case of no response to the written invitation, one reminder was sent. The individuals, who accepted the invitation, received oral information about the study, and after signing a written consent form, they were scheduled for a full-mouth radiographic survey (FMS) during 2008–09 at the Department of Dentistry and Oral Health, Section for Oral Radiology and Endodontics, Aarhus University, Aarhus, Denmark. Only individuals, who had at least one tooth, were included in the study. All participants, who attended the first examination, were again invited to participate in the second and third radiographic examination approximately 5 and 10 years after the first examination, resulting in longitudinal information based on three FMSs performed at approximately 5-year intervals (C2: 2009–2014–2019). No clinical examinations were performed. The study complies with ‘STrengthening the Reporting of OBservational studies in Epidemiology’ (STROBE) guidelines [13]. A flowchart describes the participation in C1 and C2 (Figures 1 and 2). Analyses of non-participation were previously performed for C1 and C2 [14,15]. The study design was approved by the Regional Committee of Ethics (Registration number: 1-10-72-450-17).

### Radiographic examination and registration of C2

The FMS consisted of 14 periapical and two bitewing radiographs, one in each side. The radiographic examinations were performed by trained radiographers using the standard radiographic recording tools and techniques (paralleling technique and a focus-receptor distance of 28 cm). For the first examination (baseline 2008-09), the radiographs were taken with a Gendex GX-1000 X-ray unit (Gendex Corporation, Milwaukee, Wisconsin, USA) with the exposure settings 70 kV and 10 mA. Kodak Insight films (Eastman Kodak; Rochester, NY, USA) and an automatic developing unit (Dürr 1330, AC 245 L, Bietigheim-Bissingen, Germany) were used for film processing. The radiographic images were later scanned with a flatbed transparency scanner (Epson Expression, 1680 Pro; Seiko Epson Corp., Nagano, Japan) in 266 dpi and saved in Tagged Image File Format (TIFF). For examination 2 (2014) and examination 3 (2019), digital radiographs were taken with a Soredex MinRay or KaVo Fokus X-ray unit (KaVo Dental GmbH, Biberach/Riß, Germany) with the exposure settings 60 kV and 7 mA. The images were acquired using Vista imaging phosphor plates and a VistaScan Mini Plus scanner (Dürr Dental, Bietigheim-Bissingen, Germany).

The FMSs were used to record the presence or absence of teeth (third molars excluded), AP, and root filled teeth. The presence of AP was assessed using periapical index (PAI) [17]. All assessments were performed on the tooth level. In teeth with multiple roots, the root with the highest PAI score determined the periapical status of the tooth (Table 1).

**Table 1:** Radiographic variables and diagnostic categories.

**Age group** ^b^
20–29 years
30–39 years
40–49 years
50–59 years
60–64 years
**Root filled tooth**
0 = No radiographic sign of RF detectable in any root canal of the tooth
1 = Radiographic sign of RF material detectable in the root canal of tooth
**Apical periodontitis (AP), dichotomized Periapical Index (PAI)^a^**
0 = No AP (PAI 1, PAI 2)
1 = AP present (PAI 3, PAI 4, PAI 5)
**Tooth loss/extraction**
0 = No extraction, tooth present in previous and subsequent examination	1 = Extraction, tooth present in previous and absent in subsequent examination

RF: root filling; AP: apical periodontitis.

a (Ørstavik et al. [17]).

b Age groups were defined as the five different age categories, into which the individuals in study population were categorized at the time of baseline/first examination.

### Observer calibration of C2

The radiographic assessments were performed by three observers: LLK (examination 1, 2 and 3), LJ (examination 1), and AR (examination 2 and 3). The observers were dentists with specialized training in endodontics. Thorough calibration amongst the observers was performed [11]. A calibration course for the PAI consisting of a set of 100 radiographs of root filled and non-root filled teeth was undertaken by all observers before the assessment of the periapical status in the radiographs. Each tooth was assigned a PAI score ranging from 1 to 5 by comparing the radiographs to a template of visual reference images with a corresponding scale as defined by Ørstavik [17]. The results of this score were then compared to a ‘gold standard atlas’ and Cohen’s Kappa was calculated.

Cohen’s kappa after the PAI calibration course was 0.81 for observer LLK, 0.82 for observer LJ, and 0.80 for observer AR. For observer LLK, intra-observer agreement scores gave Kappa values of 0.83 for PAI and 1.00 for the presence of a root filling [18]. For observer AR, intra-observer agreement scores obtained from rescoring 389 teeth in 15 individuals resulted in Cohen’s Kappa of 0.83 for PAI, and 1.00 for the presence of root fillings. Inter-observer agreement scores for PAI resulted in Cohen’s Kappa of 0.82 for observers LLK and LJ, and Cohen’s Kappa of 0.77 for observers LLK and AR. In case of disagreement, the case was discussed until consensus could be obtained.

### Data management and statistical methods

Data was recorded in an Excel spreadsheet and transferred to Stata version 16 for statistical analyses (StataCorp. 2019. Stata: Release 16. Statistical Software. College Station, TX: StataCorp LLC.). At baseline, individuals in C1 and C2 were categorized into five 10-year age groups, and the number and proportion of individuals, who participated in the first examination, and those, who participated in all three examinations, was determined. Descriptive analyses of the study population were performed with both the individual and the tooth as the unit of analysis. For each pattern of participation in C2, summary statistics were obtained about age, number of teeth, AP, root filled teeth, and AP in root filled teeth. For C1, a similar table appears in a previous paper [10].

Further analyses focused on the group of individuals, who participated in all three examinations in C1 and C2, respectively. The distribution in C2 of the number of AP per person at each of the three examinations was recorded and compared to the analogous distribution in C1 using logistic regression analysis. The analyses included a comparison of the proportion of individuals with no AP, and a comparison of the proportion of individuals with at least two teeth with AP. The combined data from all examinations were analyzed together using robust standard errors to account for the correlation between outcome from different examinations of the same individual. Similar analyses were performed for root-filled teeth and lost teeth.

For each participant at each examination, the proportion of teeth with AP, the proportion of root filled teeth, and the proportion of lost teeth were computed. These proportions were cross-classified on cohort and age group and the averages were plotted against age to identify effect of age on the outcome. For each individual and each outcome, two summary statistics were computed from the proportions: *a slope* computed as the proportion at the last examination minus the proportion at the first examination, and *a level* computed as the average of the three proportions. To facilitate the interpretation of the effect of age, period, and cohort on the proportions, a multiple regression analysis of each summary statistics was performed with age group, cohort, and their interaction as independent variables.

## Results

The distribution of the individuals in five age groups was described for the three examinations in C1 and C2 (Table 2). There were 330 individuals in C1 and 170 individuals in C2, who participated in all three examinations (Figures 1 and 2). The participation rate at the first examination was lower in C2 (33.2%) compared to C1 (51.4%). The follow-up participation rate in C2 was also lower than in C1, in particular for the younger age groups (Table 2). At baseline, the mean age in C2 was higher compared to C1 (*p* <.002) and the age difference was even larger for the individuals, who participated in all three examinations (mean age, C1: 42.9; C2: 47.3 years, *p* <.001).

**Table 2:** Age group distribution of individuals participating in all three examinations in C1 and C2.

C1					
Age groups	Participants in examination 1		Participants in all three examinations		Proportion of all participants (%)
	N	Proportion (%)	N	Proportion (%)	
20–29	111	18	49	15	44
30–39	154	25	86	26	56
40–49	170	28	100	30	59
50–59	145	24	79	24	54
60+	36	6	16	5	44
Total	616	100	330	100	54
C2					
Age groups	Participants in examination 1		Participants in all three examinations		Proportion of all participants (%)
	*N*	Proportion (%)	*N*	Proportion (%)	
20–29	65	16	16	9	25
30–39	82	21	28	16	34
40–49	89	22	44	26	49
50–59	109	27	52	31	48
60+	53	13	30	18	57
Total	398	100	170	100	43

C1: Cohort 1 (1997–2003–2008); C2: Cohort 2 (2009–2014–2019).

Overall, individuals, who participated in all three examinations in C2, did not differ much from individuals, who only participated in the first or the first and the second examination regarding the median number of teeth, prevalence of AP, prevalence of root filled teeth, total number of teeth, number of teeth with AP, root filled teeth, and root filled teeth with AP (Table 3). Similar observations were reported for C1 [10].

**Table 3:** Participation and distribution of individuals and teeth in 2009, 2014, and 2019 in C2.

Participants	Status 2009		Status 2014			Status 2019
	*n*	%	*n*	%	*n*	%
Participant in all three examinations						
Number of individuals	170		170		170	
Median number of teeth	28		28		28	
Individuals with AP	84	49.4	94	55.3	108	63.5
Individuals with root filled teeth	75	44.1	78	45.9	88	51.8
Number of teeth	4599		4566		4519	
Teeth with AP	168	3.7	205	4.5	230	5.1
Root filled teeth	164	3.6	178	3.9	189	4.2
Root filled teeth with AP	91	55.5	90	50.5	95	50.3
Participants in Examination 1 and 2						
Number of individuals	65		65			
Median number of teeth	27		27			
Individuals with AP	31	47.7	41	63.1		
Individuals with root filled teeth	29	44.6	31	47.7		
Number of teeth	1734		1706			
Teeth with AP	58	3.3	94	5.5		
Root filled teeth	51	2.9	57	3.3		
Root filled teeth with AP	26	51.0	31	54.4		
Participants in Examination 1 only						
Number of individuals	163					
Median number of teeth	28					
Individuals with AP	76	46.6				
Individuals with root filled teeth	75	46.0				
Number of teeth	4332					
Teeth with AP	189	4.1				
Root filled teeth	174	4.0				
Root filled teeth with AP	100	57.5				
Total						
Number of individuals	398		235		170	
Median number of teeth	28		28		28	
Individuals with AP	191	48.0	135	57.4	108	63.5
Individuals with root filled teeth	179	45.0	109	46.4	88	51.8
Number of teeth	10,665		6272		4519	
Teeth with AP	415	3.7	299	4.8	230	5.1
Root filled teeth	389	3.6	235	3.7	189	4.2
Root filled teeth with AP	217	55.8	121	51.5	95	50.3

Number and proportion of individuals, individuals with apical periodontitis (AP), individuals with root filled teeth, and the median number of teeth. Number and proportion of teeth, teeth with AP, root filled teeth, and root filled teeth with AP. C2: Cohort 2 (2009 − 2014 − 2019); AP: apical periodontitis.

**Figure 3:**
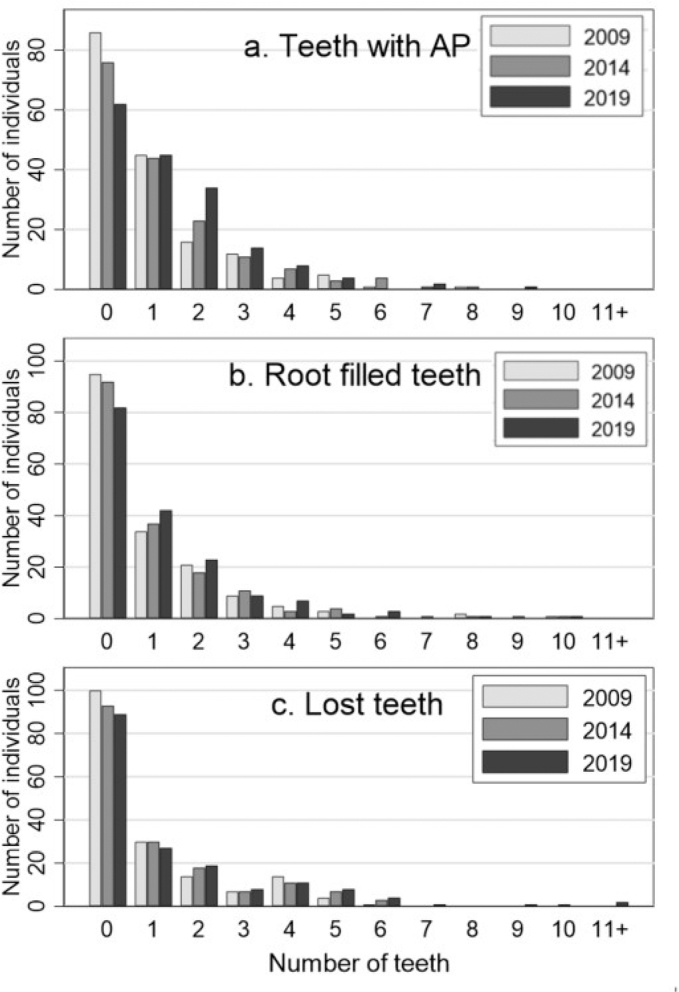
Distribution of individuals in C2 (2009, 2014, and 2019) regarding number of (a) teeth with apical periodontitis (AP), (b) root filled teeth, and (c) lost teeth*. C2: Cohort 2 (2009 − 2014 − 2019). AP: apical periodontitis. *See Kirkevang et al. [10] for corresponding information on C1: Cohort 1 (1997 − 2003 − 2008).

The number of individuals, who had no AP and no root filled teeth, decreased from 2009 to 2019 (Figure 3). For C1, a similar figure has been reported [10]. A comparison of C1 and C2 showed no statistically significant difference in the proportion of individuals with no AP, when the comparison was corrected for differences in the age distribution (*p* =.46). This was also seen for the proportion of individuals with at least two teeth with AP (*p* =.97). For root filled teeth, similar comparisons of C1 and C2 showed that C2 had a significantly higher proportion of individuals with no root fillings (*p* <.001) than C1. Also, the proportion of individuals with at least two root fillings (*p* <.001) was significantly lower in C2 than in C1. Moreover, the proportion of individuals with no lost teeth was higher in C2 (*p* =.02) and the proportion of individuals with at least two lost teeth was lower in C2 when compared to C1 (*p* <.001).

**Figure 4:**
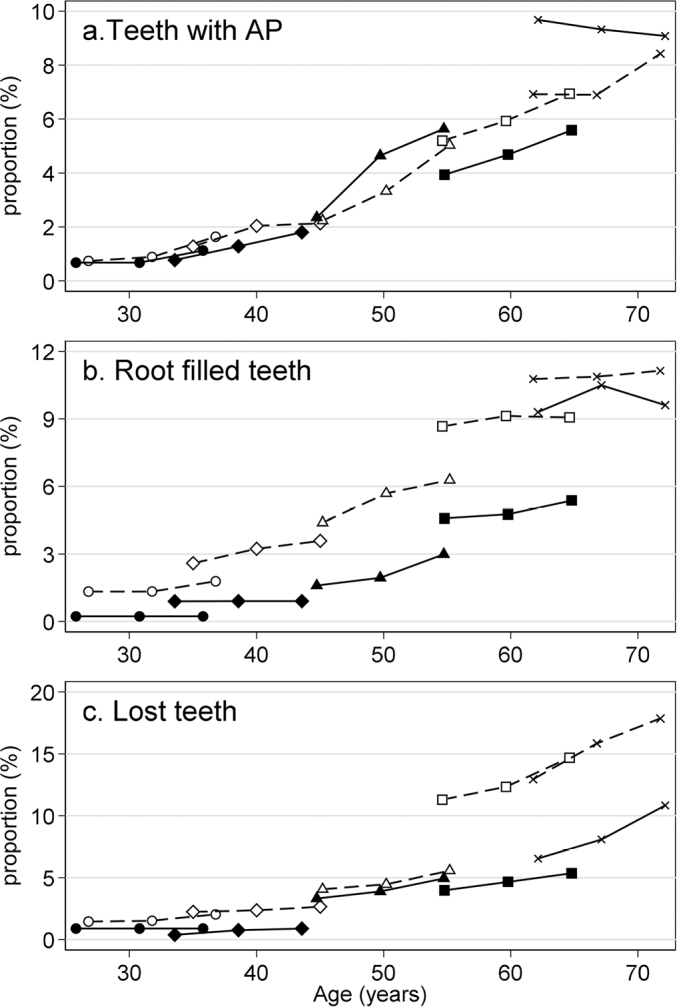
Development of proportion of (a) teeth with apical periodontitis (AP), (b) root filled teeth, and (c) lost teeth per individual in C1 (Kirkevang et al. [10]) and C2. Average proportion at each examination for each of the five age groups defined from age at baseline plotted against average age in each age group. C1: dashed lines, C2: solid lines. C1: Cohort 1 (1997 − 2003 − 2008) ; C2: Cohort 2 (2009 − 2014 − 2019); AP: apical periodontitis.

Figure 4 shows averages of the proportions of teeth with AP, root filled teeth, and lost teeth per individual for each age group at each examination. The averages are plotted against the average age in the age group at the particular examination for C1 and C2. A dashed line in C1 and a solid line in C2 connect the three average proportions from the same age group. The lines show how the proportion of outcomes change with age during a 10-year follow-up within each age group. This represents a section of the **age dependence within a cohort** and reflects the effect of age and time period on the proportion. Connecting average proportions from the same examination with lines (not shown) identify the age dependence at a particular time point. This **cross-sectional or period age dependence** reflects the effect of age and cohort on the proportions.

For the proportion of teeth with AP, the lines connecting proportions within the age groups form an increasing curve that gradually becomes steeper. This is seen for both C1 and C2, and two curves seem identical (Figure 4(a)). This description is supported by the multiple regression analyses of two summary statistics. The effect of age on the slope did not differ between the cohorts (test for no interaction: *p*=.38), the common age effect was highly significant (*p*<.001), but the slopes did not depend on cohort (*p*=.71). The analysis of the level showed similar results: no interaction between age group and cohort (*p*=.17), a strong dependence on age (*p*<.001), and no difference between the two cohorts (*p*=.65).

For the proportion of root filled teeth, the lines connecting the proportions within age groups also form an increasing curve, but for both C1 and C2 small incremental jumps are present when going from one age group to the next group. The C1 and C2 curves have the same form, but the C1 curve is clearly at a higher level than the C2 curve (Figure 4(b)). Again, the multiple regression analyses support this description. For the slope, the effect of age did not differ between C1 and C2 (*p* =.70), the dependence on age was highly significant (*p* =.006), but the slopes did not depend on cohort (*p* =0.30). The analysis of the level showed no interaction between age group and cohort (*p* =.48), a strong dependence on age (*p* <.001), and a highly significant difference between the levels of C1 and C2 (*p* <.001). Thus, the analyses showed that the proportion of root filled teeth was higher in C1 than in C2, but the dependence on age was the same.

For the proportion of tooth loss, the curves for C1 and C2 seem similar for the three youngest age groups (Figure 4(c)) and resemble what is seen for teeth with AP (Figure 4(a)). In C2, this pattern continues in the final age groups, but in C1, the proportion of lost teeth becomes much larger in the oldest age groups. The multiple regression analysis of the slope showed that the effect of age did not differ between cohorts. Moreover, the dependence on age was highly significant (*p* <.001), but the slopes did not differ between C1 and C2 (*p* =.11). The analysis of the level showed that the difference between C1 and C2 depended significantly on age group (test for no interaction: *p* =.002).

## Discussion

This longitudinal cohort study provided a comprehensive overview of time-related changes in individuals regarding teeth with AP, root filled teeth, and lost teeth in the Danish population from 2009 to 2019 and allowed a comparison with changes in a previous cohort C1 (1997–2003–2008). The study is the first of its kind to assess temporal changes in periapical and endodontic status in two similar cohorts over a 20-year period.

The present study was based on radiographic information. The general limitations of radiographic information have been discussed in a previous study [11]. In the present study, radiographic data was collected prospectively at repeated examinations performed during a period of more than 20 years. When performing studies of humans over such a long period of time, changes in radiographic modalities are inevitable, e.g. the change from conventional radiography to digital radiography. To ensure comparability of conventional radiographs from the early examinations, all traditional radiographic images were scanned in the highest possible resolution and assessed on a computer screen so that viewing conditions were as similar as possible to the digital images, which were obtained from the more recent examinations [12]. In addition, the use of several observers necessitated a thorough calibration of the observers. This has been described and discussed in detail in previous articles [11,12].

The number of participants in C2 was lower than in C1, both at baseline and at follow-up. Still, the participation rates for C1 and C2 were within the range, 40–57%, reported in previous longitudinal, observational studies [19–22]. A general trend of decreasing participation rates and a higher number of drop-outs during follow-up periods has been observed in other recent epidemiological studies, and the effect has been discussed in a paper by Galea & Tracy [23]. Low participation rates do not necessarily imply a high level of bias. On the contrary, it is the difference between the participants and non-participants that affects the amount of non-participation bias. Analyses of non-participation in the first examination for C1 and C2 were performed previously. Few differences between participants and non-participants were detected, and it was concluded that non-participation should not be a major concern [11,14,15]. The inclusion of additional participants was not attempted in the present study as it might have increased the risk of selection bias.

The descriptive analyses of individuals participating in the first, second, and third examinations indicated that the selection caused by drop-out during follow-up did not introduce serious bias for main findings regarding teeth with AP, root filled teeth, and tooth extractions in C1 and C2. A comparison of individuals, who participated in all three examinations, with those, who did not, showed that the individuals, who did not participate in all three examinations, were younger; but no differences in the number of root filled teeth per person and the proportion of root filled teeth with AP were found [12]. Thus, the results based on individuals from C1 and C2, who participated in all three examinations, seem relevant.

### Age, period, and cohort effects

Age, period, and cohort effects are epidemiological concepts rarely used in endodontics; however, they may help to describe and explain time-related changes of a particular outcome. For a specific age group followed over a 10-year period, changes may reflect both an effect of getting older (age effect) and an effect of external factors related to the period (period effect). For different age groups at a given time point, differences in outcomes may reflect both an age effect (effect of getting older) and a cohort effect (effect of time of birth).

It should be considered that external changes during the study period could have affected the results of this study. During the last 20–30 years, introduction and use of internet have led to an increased access and sharing of knowledge, which may in turn affect the oral awareness. In Denmark, the majority of the adult population have access to dental treatment primarily provided by private dental practitioners. The treatment cost is paid partly by the national health care system and partly by the patient. In 1999, a change in remuneration was introduced, allowing dentists to set individual prices on root canal treatments. This change is not expected to have affected the results in the present study, since it would have had almost similar effect on the two cohorts. Another, and perhaps more important change occurring during the observation period of the present study, was the global financial crises (2007–2009), which almost coincided with the first examination of C2. According to Statistic Denmark, the general use of dental services decreased during this and the subsequent period [12]. It indicates that the focus on oral health care was closely related to the economic situation. Oral health may also be influenced by lifestyle and systemic diseases. Recently, associations with smoking habits and type 2 diabetes mellitus have been reported [24–27]. However, these associations may not explain the present findings.

A significant development in the 1960s was the discovery of the role of fluorides in reducing caries and the subsequent introduction and use of fluoride toothpastes; cohorts born before this period may have had less protection against caries [28]. Moreover, during the 1970s routine dental visits became more common, possibly due to the sound economic situation and increased awareness of oral health [29,30].

### Teeth with AP

The age dependence of AP may be described in two different ways. The proportion of teeth with AP increases as the person gets older. Alternatively, the proportion of teeth with AP may be compared at a given time between persons with different ages. The lines in Figure 4(a) show the first type of age dependence. Connecting proportions of AP from the same examination would show the alternative age dependence. The patterns seen in C1 and C2 are remarkably similar and in both cohort, the two types of age dependence coincide, and there seemed to be no effect of change in the period concerning the number of individuals with no AP. The findings from the present study support findings from previous studies on C1 [10,11]. The occurrence of AP therefore seems to be a stable phenomenon, independent of the time period.

### Root filled teeth

The prevalence and frequency of root filled teeth increased with age, in particular in age groups above 40 years. This accumulation of root canal treated teeth over time in the individual could be expected in the present study, as the level of tooth extractions was low. Other longitudinal studies on endodontic status have reported similar findings [7,19,31,32]. This was further supported by a Swedish 20-year follow-up study, in which a high extraction rate was reported together with a lower proportion of root filled teeth with AP [21]. There were more individuals in C2, who had no root filled teeth than in C1, and especially in young age groups, fewer root filled teeth were seen in C2 than in C1. This may be related to the general improvement in oral health observed during recent decades. However, this simple explanation alone does not perfectly justify the observations for all age groups, as the proportion for the third examination in one age group in C1 did not always coincide with the proportion for the first examination in the next age group in C2.

### Tooth loss

Tooth loss typically follows from a combination of several dental problems, most often periodontal and endodontic problems combined with caries and loss of tooth structure. C2 had more individuals with no tooth loss than C1, and this may reflect a general improvement of dental health. Not surprisingly, tooth loss increased with age in both C1 and C2. In age groups below 40 years, tooth loss was similar in C1 and C2. However, the proportions of lost teeth in the two oldest age groups differ considerably between the two study populations resulting in a statistically significant interaction between age and cohort; this difference may indicate a cohort effect. It may be speculated that individuals in the oldest age groups lived the early part of their lives in a period with less focus on oral hygiene and the preventive effect of fluoride, and/or with different treatment strategies and priorities, where tooth extractions were considered an inevitable part of getting older.

### Generalizability of results

The results of the present study are relevant for the Danish adult population. Development in disease may be universal, but treatment strategies most often are also affected by politics, health care strategies, and remuneration systems, which may also affect treatment strategies, and these factors differ significantly among countries. Direct comparison among countries should therefore be cautiously interpreted. It would be interesting, though, to compare results from the present study to similar studies performed in other countries, but unfortunately, such studies are not available.

### Future perspectives

The World Health Organization (WHO) has estimated that the global population of persons older than 60 years will be doubled by 2050 [4]. A concomitant increase in the number of persons retaining more teeth may change the pattern of oral health care needs in the future. There may be an increased need for more complex and complicated dental treatments. From an endodontic point of view, this could mean more cases with local changes such as, sclerosis of root canals, or more general changes such as an increased prevalence of general diseases like diabetes that may affect healing. Furthermore, treatment challenges associated with increased frailness or dependency for very old individuals might be a concern [33]. Therefore, it seems important to perform studies including the elderly populations, and to identify changes in their dental status. Identifying challenges and barriers associated with specific birth cohorts and age groups may provide a different perspective. This may hopefully minimize the disease burden and tooth loss in the ageing population and improve the quality of life for the elderly in vulnerable groups.

## Conclusion

In C2, the prevalence of teeth with AP and root fillings increased with age, and few teeth were lost. The change in the proportion of AP was similar in two cohorts, but there were fewer root filled teeth, and fewer lost teeth in C2. The proportion of lost teeth in C2 showed a cohort effect for the oldest age groups.

## Data Availability

The data are not available due to privacy or ethical restrictions.

## Reporting guidelines

This study has been conducted in accordance with the reporting guidelines by STROBE for observational studies and the recommended checklist is enclosed.
